# Supplementary material for: An asymmetric centromeric nucleosome
Source: eLife. 2018 Aug 23;7:e37911. doi: 10.7554/eLife.37911 (PMC6125124; doi:10.7554/eLife.37911)
Supplement: Supplementary file 1. [file elife-37911-supp1.pdf]

Table S1. Strain list

Strain number	Description	Parent
PKY2160	*MATa; leu2-3, 112; ura3-1; his3-11,15; trp1-1; ade2-1; can1-100;*<br>*cse4::kanMX; pcse4-107-HA-URA3*	W303 background (Sharp et al. 2002).
PKY5394	+ pRS413 = (*HIS3, CEN/ARS*)	PKY2160
PKY5230<br>(Wild-type)	+ pPK796 = (*CSE4 wild type*, *LEU2, CEN/ARS*)<br>+ pRS414 = (*TRP1, CEN/ARS*)	PKY2160
PKY5232<br>(Chimera)	+ pPK819 = (*cse4 (G196S, L198F, H200D, L204A, L206I, I224L)*, *LEU2, CEN/ARS*)<br>+ pRS414 = (*TRP1, CEN/ARS*)	PKY2160
PKY5234<br>(X + Y)	+ pPK820 = (*cse4 (G196S, L198F, H200D, L204A, L206I, L220A, I224V)*, *LEU2, CEN/ARS*)<br>+ pPK815 = (*cse4 (G196S, L198F, H200D, L203I, L204W, L206I), TRP1, CEN/ARS*)	PKY2160
PKY5470<br>(Wild-type)	+ pPK796 = (*CSE4 wild type*, *LEU2, CEN/ARS*)<br>+ pRS414 = (*TRP1, CEN/ARS*)<br>+ pRS413 = (*HIS3, CEN/ARS*)	PKY5394
PKY5473<br>(Chimera)	+ pPK819 = (*cse4 (G196S, L198F, H200D, L204A, L206I, I224L)*, *LEU2, CEN/ARS*)<br>+ pRS414 = (*TRP1, CEN/ARS*)<br>+ pRS413 = (*HIS3, CEN/ARS*)	PKY5394
PKY5476<br>(X + Y)	+ pPK820 = (*cse4 (G196S, L198F, H200D, L204A, L206I, L220A, I224V)*, *LEU2, CEN/ARS*)<br>+ pPK815 = (*cse4 (G196S, L198F, H200D, L203I, L204W, L206I), TRP1, CEN/ARS*)<br>+ pRS413 = (*HIS3, CEN/ARS*)	PKY5394
PKY5485<br>(Δ70X + Y)	+ pPK948 = (*cse4 (Δ1-70, G196S, L198F, H200D, L204A, L206I, L220A, I224V)*, *LEU2, CEN/ARS*)<br>+ pPK815 = (*cse4 (G196S, L198F, H200D, L203I, L204W, L206I), TRP1, CEN/ARS*)<br>+ pRS413 = (*HIS3, CEN/ARS*)	PKY5394
PKY5488<br>(X + Δ70Y)	+ pPK820 = (*cse4 (G196S, L198F, H200D, L204A, L206I, L220A, I224V)*, *LEU2, CEN/ARS*)<br>+ pPK949 = (*cse4 (Δ1-70, G196S, L198F, H200D, L203I, L204W, L206I), TRP1, CEN/ARS*)<br>+ pRS413 = (*HIS3, CEN/ARS*)	PKY5394
PKY5491<br>(Cse4 ERS)	+ pPK957 = (*cse4 (Q227E, F228R, I229S)*, *LEU2, CEN/ARS*)<br>+ pRS414 = (*TRP1, CEN/ARS*)<br>+ pRS413 = (*HIS3, CEN/ARS*)	PKY5394
PKY5494<br>(Chimera ERS)	+ pPK953 = (*cse4 (G196S, L198F, H200D, L204A, L206I, I224L, Q227E, F228R, I229S)*, *LEU2, CEN/ARS*)<br>+ pRS414 = (*TRP1, CEN/ARS*)<br>+ pRS413 = (*HIS3, CEN/ARS*)	PKY5394

PKY5497<br>(X ERS + Y)	+ pPK951 = (*cse4 (G196S, L198F, H200D, L204A, L206I, L220A, I224V, Q227E, F228R, I229S)*, *LEU2, CEN/ARS*)<br>+ pPK815 = (*cse4 (G196S, L198F, H200D, L203I, L204W, L206I), TRP1, CEN/ARS*)<br>+ pRS413 = (*HIS3, CEN/ARS*)	PKY5394
PKY5500<br>(X + Y ERS)	+ pPK820 = (*cse4 (G196S, L198F, H200D, L204A, L206I, L220A, I224V)*, *LEU2, CEN/ARS*)<br>+ pPK952 = (*cse4 (G196S, L198F, H200D, L203I, L204W, L206I, Q227E, F228R, I229S), TRP1, CEN/ARS*)<br>+ pRS413 = (*HIS3, CEN/ARS*)	PKY5394
PKY5503<br>(X ERS + Y ERS)	+ pPK951 = (*cse4 (G196S, L198F, H200D, L204A, L206I, L220A, I224V, Q227E, F228R, I229S)*, *LEU2, CEN/ARS*)<br>+ pPK952 = (*cse4 (G196S, L198F, H200D, L203I, L204W, L206I, Q227E, F228R, I229S), TRP1, CEN/ARS*)<br>+ pRS413 = (*HIS3, CEN/ARS*)	PKY5394
PKY5506<br>(Cse4 L1-ERS)	+ pPK969 = (*cse4 (Δ172-174, W178F, Q227E, F228R, I229S)*, *LEU2, CEN/ARS*)<br>+ pRS414 = (*TRP1, CEN/ARS*)<br>+ pRS413 = (*HIS3, CEN/ARS*)	PKY5394
PKY5509<br>(X L1-ERS + Y)	+ pPK966 = (*cse4 (Δ172-174, W178F, G196S, L198F, H200D, L204A, L206I, L220A, I224V, Q227E, F228R, I229S)*, *LEU2, CEN/ARS*)<br>+ pPK815 = (*cse4 (G196S, L198F, H200D, L203I, L204W, L206I), TRP1, CEN/ARS*)<br>+ pRS413 = (*HIS3, CEN/ARS*)	PKY5394
PKY5512<br>(X + Y L1-ERS)	+ pPK820 = (*cse4 (G196S, L198F, H200D, L204A, L206I, L220A, I224V)*, *LEU2, CEN/ARS*)<br>+ pPK967 = (*cse4 (Δ172-174, W178F,G196S, L198F, H200D, L203I, L204W, L206I, Q227E, F228R, I229S), TRP1, CEN/ARS*)<br>+ pRS413 = (*HIS3, CEN/ARS*)	PKY5394
PKY5515<br>(Δ70X L1-ERS<br>+ Y)	+ pPK964 = (*cse4 (Δ1–70, Δ172-174, W178F, G196S, L198F, H200D, L204A, L206I, L220A, I224V, Q227E, F228R, I229S)*, *LEU2, CEN/ARS*)<br>+ pPK815 = (*cse4 (G196S, L198F, H200D, L203I, L204W, L206I), TRP1, CEN/ARS*)<br>+ pRS413 = (*HIS3, CEN/ARS*)	PKY5394
PKY5518<br>(X<br>+ Δ70Y L1-ERS)	+ pPK820 = (*cse4 (G196S, L198F, H200D, L204A, L206I, L220A, I224V)*, *LEU2, CEN/ARS*)<br>+ pPK965 = (*cse4 (Δ1–70, Δ172-174, W178F,G196S, L198F, H200D, L203I, L204W, L206I, Q227E, F228R, I229S), TRP1, CEN/ARS*)<br>+ pRS413 = (*HIS3, CEN/ARS*)	PKY5394
PKY5521<br>(X L1-ERS	+ pPK966 = (*cse4 (Δ172-174, W178F, G196S, L198F, H200D, L204A, L206I, L220A, I224V, Q227E, F228R, I229S)*, *LEU2, CEN/ARS*)	PKY5394

+ Δ70Y)	+ pPK949 = (*cse4 (Δ1–70, G196S, L198F, H200D, L203I, L204W, L206I), TRP1, CEN/ARS*)<br><br>+ pRS413 = (*HIS3, CEN/ARS*)	
PKY5524<br><br>(Δ70X<br><br>+ Y L1-ERS)	+ pPK948 = (*cse4 (Δ1–70, G196S, L198F, H200D, L204A, L206I, L220A, I224V), LEU2, CEN/ARS*)<br><br>+ pPK967 = (*cse4 (Δ172-174, W178F,G196S, L198F, H200D, L203I, L204W, L206I, Q227E, F228R, I229S), TRP1, CEN/ARS*)<br><br>+ pRS413 = (*HIS3, CEN/ARS*)	PKY5394
